# [Corrigendum] Decreased expression of CD63 tetraspanin protein predicts elevated malignant potential in human esophageal cancer

**DOI:** 10.3892/ol.2026.15882

**Published:** 2026-09-24

**Authors:** Xiaojing Lai, Qing Gu, Xia Zhou, Wei Feng, Xiao Lin, Yan He, Jinming Cao, Pengfei Liu, Huojun Zhang, Xiao Zheng

Oncol Lett 13: 4245–4251, 2017; DOI: 10.3892/ol.2017.6023

Subsequently to the publication of the above article, an interested reader drew to the Editor's attention that, concerning the cellular invasion assay data shown in Fig. 4B on p. 4249, the ‘Control’ and ‘‘NC’ data panels were apparently matching, suggesting that this figure had been assembled incorrectly.

After re-examining their original data, the authors acknowledged that this figure had been compiled incorrectly. However, they were able to consult their original data, and the corrected version of Fig. 4, now showing data for Fig. 4B from an alternative experiment, is shown on the next page. Note that the errors made in assembling this figure did not significantly affect either the results or the conclusions reported in this study. The authors are grateful to the Editor of *Oncology Letters* for allowing them this opportunity to publish a Corrigendum, and all the authors agree with its publication. Furthermore, the authors apologize to the readership for any inconvenience caused.

## Figures and Tables

**Figure 4.**
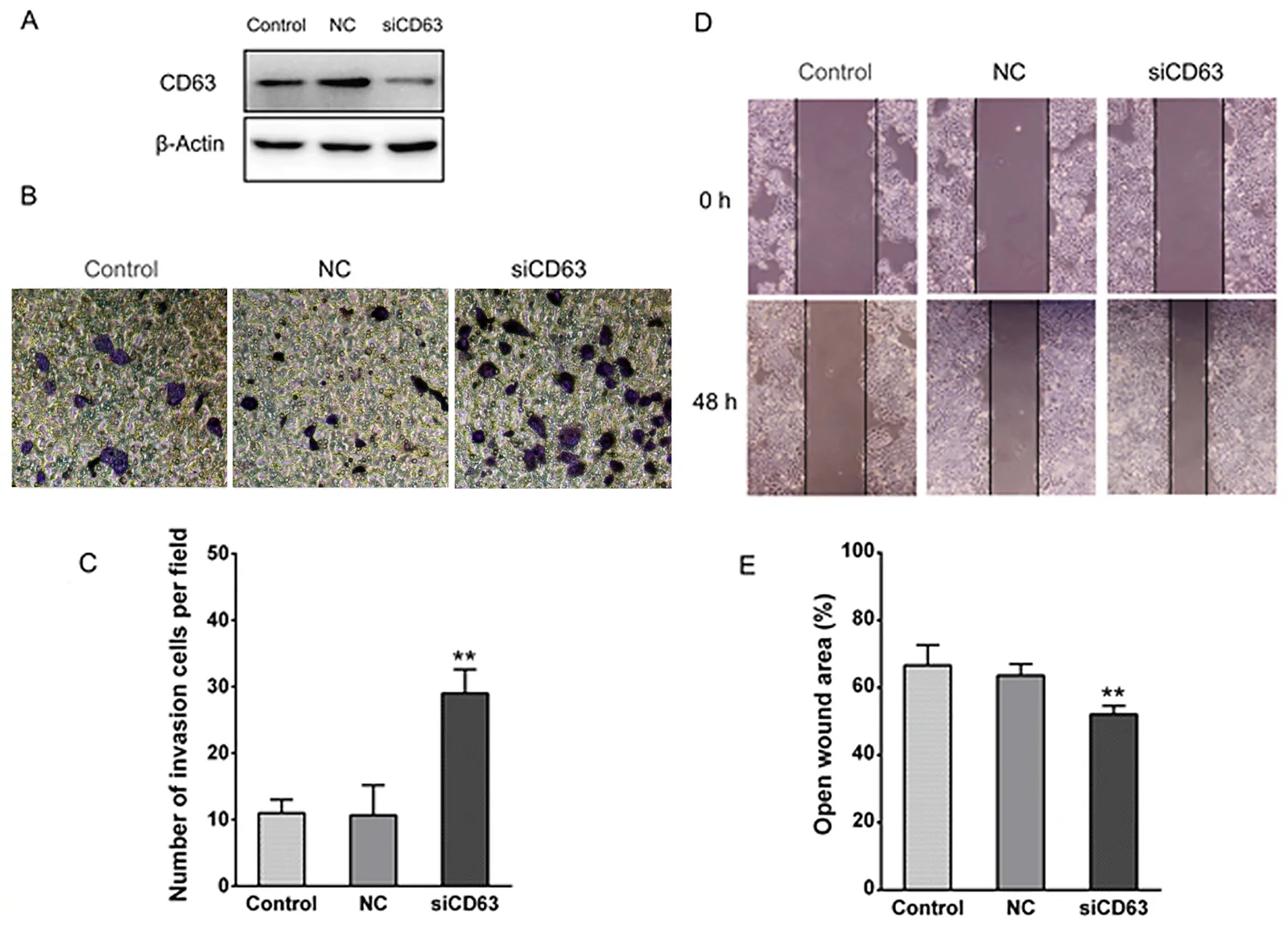
Effect of CD63 on esophageal cancer cell invasiveness. (A) Western blot analysis confirmed a marked reduction of CD63 protein following CD63 small interfering RNA transfection in esophageal cancer cells. (B and C) In the Matrigel invasion assay, following treatment for 48 h, the number of CD63 knockdown TE-1 cells that migrated to the bottom surface of the membrane was greater compared with TE-1 cells transfected with empty plasmid and the control TE-1 cells. (D and E) Wound healing was observed 48 h after treatment, and the open wound area of CD63 knockdown TE-1 cells was larger than the TE-1 cells transfected with empty plasmid and the control TE-1 cells. **P<0.01. NC, negative control; siCD63, small interfering RNA targeting CD63.

